# Perception and Attitude of Surgical Trainees in Nigeria to Trauma Care

**DOI:** 10.1155/2021/6584813

**Published:** 2021-01-30

**Authors:** Onyedika Okoye, Emmanuel Ameh, Emmanuel Ojo

**Affiliations:** ^1^Department of Surgery, National Hospital Abuja, Abuja, Nigeria; ^2^Department of Surgery, Jos University Teaching Hospital, Jos, Nigeria

## Abstract

**Background:**

Trauma is still the leading cause of death in individuals between the ages of 1 and 44 years. Establishment of good trauma centres and systems has been shown to have a significant positive impact on outcomes. Surgical specialties, particularly trauma, are becoming less attractive in different parts of the world for a variety of reasons.

**Aim:**

The aim of this study is to ascertain the perception and attitude of future surgeons towards trauma care in Nigeria. *Materials and methods*. This is a cross-sectional study using a pretested, structured, paper-based questionnaire which was administered to consecutive surgical trainees at the annual revision course of West African College of Surgeons. Data were analyzed using SPSS version 12, and results are presented in tables and figures.

**Results:**

One hundred and fifty-seven questionnaires were adequately completed with a male-to-female ratio of 18 : 1 and median age of 30 years. There is a general agreement among the respondents that trauma incidence in Nigeria is high or very high. While about 70% of the respondents believe that the Nigerian trauma system is poorly planned, about 19% think it is nonexistent. 81 (53.7%) agree or strongly agree that managing trauma patients is too stressful. A good number, 116 (74.4%), strongly agree that having a separate dedicated trauma unit will improve care and outcome. While 82% of the surgical trainees support post fellowship training in trauma, only 62.2% will like to have the training. There is no significant difference between the proportion of males and females who would like to have the training.

**Conclusion:**

Surgical trainees in Nigeria have good perception and positive attitude towards trauma care. Primary prevention measures must be emphasized during surgical trainees' training in trauma.

## 1. Introduction

Trauma is still the leading cause of death in individuals between the ages of 1 and 44 years, and it is the third most common cause of death regardless of age [1]. Motor vehicular crashes in addition to other forms of trauma have become a major health problem throughout the world and especially in low- and middle-income countries (LMICs) [2–4]. Fast motorization and other factors contribute to the rapid increase in trauma morbidity and mortality seen in these developing countries [5]. Over five million people die per year as a consequence of trauma [6]. Trauma incidence in Nigeria is rising, accounting for half of all surgical emergencies with the greatest impact on the economically productive male adults [7, 8]. Establishment of good trauma centres and systems has been shown to have a significant positive impact on outcomes [1, 9, 10].

Despite good trauma systems in many parts of the world, approximately 50% of trauma deaths occur within seconds to minutes and these deaths must be addressed by improved injury prevention and control strategies [11]. Emphasis on trauma prevention is part of the effective trauma system. Perhaps, one of the most studied trauma prevention strategies using epidemiological tool was developed by William Haddon [12]. Although surgical specialties including trauma are becoming less attractive in different parts of the world [13–16], there are still some positive attitude and interest in trauma by surgical trainees, doctors, and medical students in some other studies across the globe [17, 18]. The aim of this study is therefore to ascertain the perception and attitude of future surgeons towards trauma care in Nigeria.

## 2. Materials and Methods

### 2.1. Study Design

This is a cross-sectional questionnaire-based survey.

### 2.2. Study Participants

The study participants were surgical trainees (registrars and senior registrars) attending the annual revision course of the West African College of Surgeons from 6 to 9 September, 2017. The participants were from fourteen different surgical training institutions across the country. Informed consent was obtained at the time of registration before administering the questionnaire.

### 2.3. Sample Technique

All the consecutive surgical trainees who registered for the course were recruited into the study at the point of registration after giving informed consent.

### 2.4. Study Instrument

A pretested, structured, paper-based, self-administered questionnaire was used. The fifteen-item questionnaire has the following parameters: biographic data such as age and sex, year of training, level of training, questions on perception of prehospital and in-hospital trauma care, trauma training and rotation, trauma calls, and stress of managing trauma patients as well as specific interest in trauma and compulsory trauma rotation.

### 2.5. Data Analysis

Data were analyzed using statistical package for the social sciences (SPSS) version 12. A test of significance was done using chi square and Student's *t*-test for qualitative and quantitative variables, respectively. Level of statistical significance was set at 95% confidence interval and *p* < 0.05. Frequency tables and charts were used where necessary to present the results.

### 2.6. Ethical Consideration

Ethical approval was obtained from the National Hospital Abuja Institution review board.

## 3. Results

**Figure 1:**
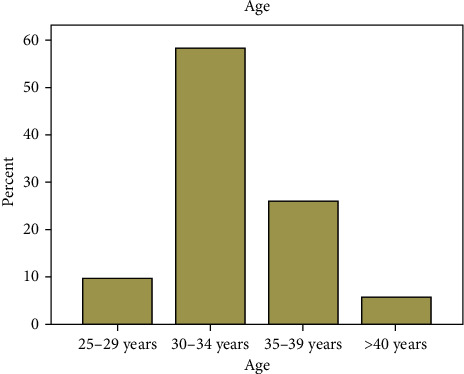
Age distribution of the respondents.

A total of 218 questionnaires were administered. Nine were incompletely filled. However, only 157 were adequately completed and returned with a response rate of 72.02%. There were 144 (94.7%) males and 8 (5.3%) females, aged 28 to 40 years with median age of 30 years (Figure 1). Second-year trainees accounted for the majority of the respondents (59.9%) while first-year trainees accounted for the minority (3.2%).

There is a general agreement that trauma incidence in Nigeria is high or very high (98.7%). Less than 1% think that the incidence is low. About half of the respondents (49.7%) have worked in a trauma centre, 66.9% believe that the trauma system in Nigeria is poorly planned, and 18.8% of the respondents think that it is nonexistent. Furthermore, prehospital care was perceived to be poor by most (91.6%) of the studied group.

While 75% of the respondents strongly agreed that every hospital should have a separate trauma unit, only 38.5% strongly agree that the same should be applicable for dedicated trauma centres. Quite a good number of the studied group 116(74.4%) strongly agreed that having separate dedicated trauma unit will improve care and outcome; only 0.6% disagree on this. Less than half of the group (43.9%) strongly agreed that trauma rotation should be made compulsory at part 2 fellowship level, and only 2.5% strongly disagreed. While nearly half of the respondents (54%) strongly agreed that trainees on trauma call should sleep in, only about a quarter (29.5%) strongly agreed the same for the consultants on call (see Table 1).

**Figure 2:**
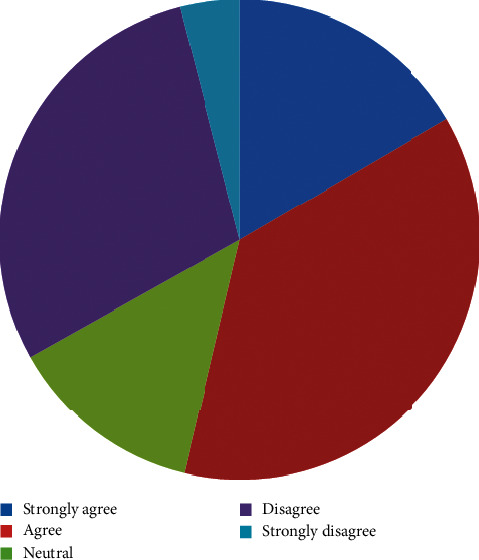
Managing trauma patients is too stressful.

Figure 2 shows that 56 (37.1%) of the participants agreed that managing trauma patients is too stressful, 25(16.6%) strongly agreed, 44(29.1%) disagree, and 6 (4.0%) strongly disagree. The proportion of the female (0.0%) who strongly agreed to this is comparatively less than their male counterpart (16.6%) though this observed difference is not statistically significant (*P*=0.346)

**Figure 3:**
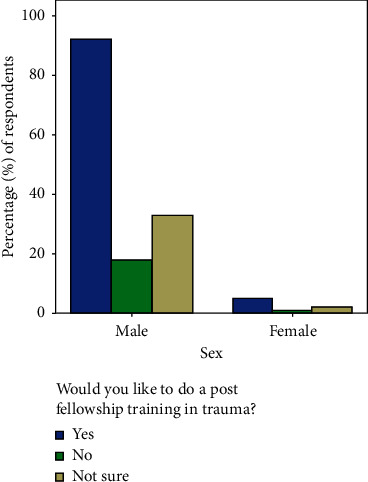
Respondents' interest in post fellowship training in trauma.

In Figure 3, while 127 (82%) of the respondents support post fellowship training in trauma, only 62.2% will like to do the training. Similarly, 62.5% of the females will like to do the training compared to 64.3% of the males. This difference is also statistically insignificant (*p*=0.992). Majority of the respondents 150 (96.2%) believe that dedicated trauma units are necessary in Nigeria, and 116 (74.8%) think that the present number is very low for the population.

**Figure 4:**
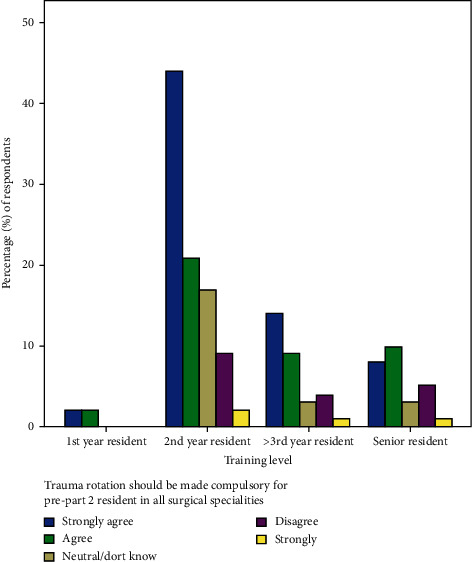
Compulsory trauma rotation for part 2 trainees.

Figure 4 shows that half (50%) of the new surgical intakes strongly agree that trauma rotation should be made compulsory for pre part 2 trainees in all surgical specialties. However, only 29.6% of the senior residents feel the same. There are variable agreement among the second-year and third-year trainees.

Finally, a very good number, 146 (94.2%), of respondents perceive trauma mortality as high, 85 (54.8%), or very high, 61 (39.4%), and the majority, 139 (89.7%), advocated primary preventive strategy.

## 4. Discussion

In this study, the male-to-female ratio of 18 : 1 reflects the low enrolment of females in surgical specialties. This may be due to the perceived stress associated with surgical residency training programme in general. This is supported by the fact that single male gender was statistically associated with the choice of surgical specialty among medical students and doctors [19]. In addition, lifestyle issues and domestic demand among others were identified as a reason for low female enrolment in surgery in another study [20]. The modal age group of 30 to 34 years accounted for 58.4% of the respondents. Although residency training programme does not have any age limit, it is mainly embarked upon by young adults as demonstrated in the result. No respondent was less than 25 years due to the long medical school duration (minimum of six years) with additional two years for internship and youth service before one is eligible to commence residency programme in Nigeria.

The fact that over 98% of the respondents agreed that trauma incidence in Nigeria is high or very high and over 90% perceived prehospital care as being poor is part of demonstration of knowledge in trauma epidemiology. Other studies have supported this high incidence of trauma in Nigeria [7, 8]. Only 38.5% of respondents strongly agreed that every hospital should have a separate trauma centre. This low response is likely related to the high cost of setting up a trauma centre which is not feasible for all the hospital in a developing economy. Nearly all the respondent (99.4%) agree or strongly agreed with about three quarter (74.4%) of the respondents strongly agreeing that separate dedicated trauma unit will improve care and outcome. This finding affirms the previous studies that have demonstrated a positive impact on outcome from establishment of the trauma system and trauma centres [1, 9, 10].

Surgical trainees on call sleep in the hospital routinely. This relatively low affirmative response for trainees on trauma call to sleep in the hospital (54.2%) is surprising and cannot be explained by common reasoning. Perhaps, the response is indirectly in favour of shifts in trauma unit call which was not assessed in this study. Further study is needed to clarify this assumption. It is not surprising, however, that only about a quarter (29.5%) of the respondents strongly agreed that trauma consultants should sleep in the hospital when on call. This may appear abnormal to the respondents since consultants are generally known to take calls from home in Nigeria.

Approximately, half of the respondents (53.7%) perceive trauma care as being too stressful. This is not different from another study where trauma care is seen as stressful and time consuming [17]. Furthermore, 70% of surgical trainees in a related study rated surgical residency programme stress as moderate to severe and attributed it to lack of facilities and unstructured nature of the programme [21]. However, the known global high incidence of trauma which is rapidly rising in the developing world may be contributory to this perceived stress. Surprisingly, none of the females (0%) strongly agreed to this stress perception as opposed to 16.6% of the males, but the difference is not significant (*p*=0.346). The small female sample size may be responsible for this lack of significance.

Surgeons in training are known to resist any action that will increase the duration of the training programme. As such, less than half (43.9%) of the respondents strongly agreed that trauma rotation should be made compulsory for part 2 trainees. Only 29.6% of senior trainees strongly agreed to this as against 50% of first year trainees. This low response from senior trainees confirms earlier inference since this subgroup will be directly affected by any extension of their training. This difference is not statistically significant (*p*=0.992).

Quite a good number of the respondents (82%) support post fellowship training in trauma. This underscores the importance of this training. Similarly, up to 62.2% of the trainees would like to do the training. This positive finding is similar to the findings in New Zealand where 76% of trainees showed interest in trauma [22] but sharply contrasts from the findings in a Canadian study where only few surgeons are truly committed to providing trauma care and one-third wished to treat no trauma patient at all [16]. Furthermore, only 5% of surgeons want greater than 30% of trauma related practice in another study [15]. This perceived low interest in trauma specialty has been grossly highlighted in other studies in different parts of the world including the United States of America [23–25]. Interestingly, nearly equal proportion of males (64.3%) and females (62.5%) will like to do trauma training in this study. This observation can be said to be grossly biased as the number of female respondents is quite low.

Dedicated trauma centres are necessary in Nigeria according to 96.2% of respondents. A large number (74.8%) think that the existing trauma centres are very few. This response is expected in view of the huge population of Nigeria which is over two hundred million. Majority (94.2%) of the respondents agree that trauma mortality is high. This is in line with existing facts in the developing countries where trauma morbidity and mortality is high [5]. Primary prevention is generally known to be the best preventive strategy, and this was advocated by up to 89.9% of the surgical trainees.

### 4.1. Limitations

The paper-based method of the questionnaire may have been responsible for the low response rate which consequently reduced the sample size. There is significant imbalance in the number of respondents at different levels of training making a suitable sampling technique difficult. The number of female participants in this study is quite low as well, making some interpretation difficult.

### 4.2. Recommendations

A web-based study will be a better alternative in this group of respondents. Prehospital trauma prevention education should be targeted at road safety corps members, national youth service corps members, journalists, and road transport association members in order to mitigate this poor perception of prehospital care in the society.

## 5. Conclusion

Surgical trainees in Nigeria have a good perception and positive attitude towards trauma care. Prehospital care was perceived to be poor by most of the trainees. Primary prevention measures must be emphasized during surgical trainees' training in trauma.

## Figures and Tables

**Table 1:** Some of the perceptions among the study group.

S/N	Survey question	Strongly agree	Agree	Neutral	Disagree	Strongly disagree
		N (%)	N (%)	N (%)	N (%)	N (%)
1	*Every hospital should have a separate trauma care unit*	117 (75)	32 (20.5)	3 (1.9)	4 (2.6)	0 (0)
2	*Every hospital should have a dedicated trauma centre*	60 (38.5)	52 (33.3)	15 (9.6)	28 (17.9)	1 (0.6)
3	*Trauma training is beneficial to pre part 2 surgical trainees in all specialties*	93 (60)	53 (34.2)	4 (2.6)	5 (3.2)	0 (0.6)
4	*Trauma rotation should be made compulsory for pre part 2 surgical trainees in all specialties*	68 (43.9)	42 (27.1)	23 (14.8)	18 (11.6)	4 (2.6)
5	*Trainees on trauma posting should sleep in the hospital when on call*	84 (54.2)	60 (38.7)	8 (5.2)	3 (1.9)	0 (0)
6	*Trauma surgeons (consultants) should sleep in the hospital when on call*	46 (29.5)	57 (36.5)	34 (21.8)	18 (11.5)	1 (0.6)
7	*Separate dedicated trauma unit will improve trauma care and outcome*	116 (74.4)	37 (23.7)	2 (1.3)	1 (0.6)	0 (0)

## Data Availability

The original data are available and can be provided whenever they are needed.
